# Serum biochemical profiles are distinct between White Leghorn chicken lines selected for divergent antibody response to sheep red blood cells

**DOI:** 10.1186/s12917-025-05277-8

**Published:** 2026-01-13

**Authors:** Anna L. Facchetti V. Assumpcao, Valentina Caputi, Christopher M. Ashwell, Christa F. Honaker, Paul B. Siegel, Robert L. Taylor, Joshua M. Lyte

**Affiliations:** 1 Department of Poultry Science, University of Arkansas, Fayetteville, AR 72701 United States of America; 2 Poultry Production and Product Safety Research Unit, Agricultural Research Service, Department of Agriculture, Fayetteville, 72701 AR United States of America; 3 School of Agriculture and Food Systems, West Virginia University, Morgantown, WV26506 United States of America; 4 School of Animal Sciences, Virginia Polytechnic Institute and State University, Blacksburg, VA 24061 United States of America

**Keywords:** Antibody, Chicken, Immunity, Immunoglobulin, Serum biochemistry

## Abstract

**Background:**

For forty-nine generations, White Leghorn chickens have been selected for divergent responses to injection of sheep red blood cells (SRBCs), generating the high (HAS) or low (LAS) antibody response lines. The objective of this study was to determine if selection for systemic antibody concentrations would result in divergence in blood serum biochemistry profiles.

**Materials and methods:**

Blood serum samples were collected from the same birds at 4, 8, 12, 16, and 66 weeks to analyze several biochemical serum parameters, including total protein (TP), albumin, globulin, albumin: globulin (A: G) ratio, aspartate transferase (AST), creatine kinase (CK), uric acid, glucose, calcium, phosphorus, potassium, sodium, total bile acid (TBA), gamma-glutamyl transferase (GGT), alanine transferase (ALT), and total cholesterol (TC).

**Results:**

Our results showed that HAS chickens had higher globulin and potassium levels and lower albumin: globulin (A: G) ratio serum concentrations than LAS chickens at 8, 12, and 16 weeks. At 12 weeks, HAS had total protein (TP) higher than LAS. Additionally, at 16 and 66 weeks, HAS had a higher concentration of creatine kinase (CK) than LAS. At 8 weeks, alanine transferase (ALT) levels were lower in HAS than LAS, and at 12 weeks, LAS females’ ALT levels were higher than HAS females. Furthermore, HAS had higher gamma-glutamyl transferase (GGT) levels than LAS at 12 and 16 weeks. The TP, globulin, and A: G ratios are consistent with the selection of HAS for higher antibody concentration. Additionally, our data also showed a divergence in potassium and liver enzyme levels between HAS and LAS. Females at 66 weeks had a biochemistry profile consistent with egg production, including increased total cholesterol (TC), total bile acid (TBA), aspartate transferase (AST), calcium, and phosphorus levels, as well as decreased uric acid and GGT concentrations compared with their male counterparts.

**Conclusions:**

Our findings demonstrate that direct selection on the humoral immune system resulted in distinct serum biochemical profiles. These results are likely to serve as potential informative and diagnostic markers in poultry health, food safety, and performance.

## Background

Divergent selection in animal populations provides a powerful tool for investigating targeted traits, such as the immune system, which can lead to changes in other physiological characteristics, such as the gut microbiome. Beginning with a common founder population, White Leghorn chickens have been divergently selected for high antibody-selected (HAS) and low antibody-selected (LAS) responses against sheep red blood cells (SRBC), over forty-nine generations. The divergent lines provide a valuable model for avian immunology [1–4]. Previous research has identified differences that help explain the phenotype, including their major histocompatibility (MHC) B-haplotypes, as well as other genetic loci identified via pooled resequencing and quantitative trait loci mapping of an advanced intercross [4–6]. While these lines have been extensively characterized in terms of immune-related gene and protein expressions and gut microbiome variations [2, 4, 7], less is known about how the divergent selection may affect their systemic physiology, including blood serum biochemistry.

Profiling blood serum biochemistry provides an integrative measure of metabolic status, organ function, and homeostatic balance. The measurement of organ-specific enzymes, metabolites, and electrolytes present in the serum can reflect the performance and physiological status of multiple organs, such as the liver, kidney, pancreas, and muscle, as well as protein and lipid turnover and energy allocation [8, 9]. The immune system has a high energy demand, causing shifts in protein, lipid, and carbohydrate metabolism [10]. The prolonged selection for high or low antibody production could cause changes in the biochemical profile, revealing how immune selection influences other physiological functions. Previous studies with HAS and LAS chickens have shown that the HAS line has lower body weight, delayed sexual maturity, and different reproductive traits than LAS [1, 11], suggesting that HAS allocate more nutrients and energy to their immune system. Additionally, whole-genome studies show divergence across ~ 20% of the genome in HAS compared to LAS lines, spanning immune and metabolic loci [6].

The immune system plays an important role in shaping the gut microbiome through several mechanisms, including mucosal antibody secretion and cytokine signaling. Moreover, the gut microbiome is a key regulator of the immune system through the co-evolution of the commensal microbes and their host [12–14], and influences the host humoral response to vaccines [15, 16]. In poultry, differences in the gut microbiome have been associated with modified bile acid metabolism and circulating biochemical markers [17, 18]. The divergent selection in the antibody production of HAS and LAS lines resulted in correlated responses in gut microbiome [2, 7], which could have contributed to the alteration of their blood serum biochemical profiles [19]. Our hypothesis proposed that selection pressure for divergent antibody response between HAS and LAS lines also generated separation of serum biochemistry profiles. The objective of this study was to determine whether selection for systemic antibody responses produced divergence in blood serum biochemistry profiles.

## Materials and methods

### Chickens

The research was conducted in two phases, and procedures were approved by the Institutional Animal Care and Use Committees at West Virginia University for 4- to 16-week-old chickens (Phase 1) and Virginia Tech for 66-week-old chickens (Phase 2). Eggs from the forty-ninth generation of the HAS and LAS lines that had undergone long-term divergent selection for antibody response to SRBC were incubated and hatched. Phase 1: chicks were randomly allocated and maintained in battery cages (10 per cage). The room was maintained at 95 °F for the first week, after which the temperature was reduced 5 °F per week until reaching 70 °F. Phase 2: at hatch, chicks were wing banded, vaccinated for Marek’s Disease, and transferred to 3.72 m^2^ concrete floor pens with wood shavings for litter and hot air brooding with a heat lamp supplement. In week 4, sex was determined by visual inspection, and rearing continued in the same pens with birds separated by sex. In week 21, chickens were transferred to individual cages with wire floors and a 14-hour light-dark photoperiod. For the duration of this study, the birds utilized were not euthanized; blood samples were simply collected from each bird and then the bird was returned to its colony. A corn-soy mash diet that met or exceeded all nutritional requirements (Starter mash 0–8 weeks: 20.9% Crude Protein, 2650 kcal/kg ME; Developer mash 8–21 weeks: 15.6% Crude Protein, 2800 kcal/kg ME; Breeder mash 21–66 weeks: 17.5% Crude Protein, 2900 kcal/kg ME), and water was provided *ad libitum* throughout the study.

### Blood serum collection

Blood samples were collected from the wing vein of the HAS and LAS birds that were maintained at Virginia Tech and West Virginia University into vacutainer tubes with a clot activator (Catalog # BD367812, BD). For phase 1, blood samples were collected from the same birds at 4, 8, 12, and 16 (*n* = 8–10 chickens/line/sex/timepoint), and for phase 2, blood samples were collected at week 66 of age (*n* = 10–12 chickens/line/sex). After collection, blood samples were centrifuged at 1100 × g for 10 min at 4 °C. Then, separated serum was transferred into 1.5 mL microcentrifuge tubes (Catalog #: 10025-726, VWR) and stored at -20 °C until analyses.

### Blood serum biochemistry analysis

Serum samples were analyzed using the MicroChem II veterinary chemistry analyzer (MicroVet Diagnostics) utilizing the Avian rotors (Catalog # 41M300, MicroVet Diagnostics) for quantification of total protein (TP) (g/dL), albumin (g/dL), globulin (g/dL), albumin: globulin (A: G) ratio, aspartate transferase (AST) (U/L), creatine kinase (CK) (U/L), uric acid (mg/dL), glucose (mg/dL), calcium (mg/dL), phosphorus (mg/dL), potassium (mmol/L), and sodium (mmol/dL). Liver rotors (Catalog # 41M260, MicroVet Diagnostics) were used to analyze total bile acid (TBA) (µmol/dL), gamma-glutamyl transferase (GGT) (U/L), alanine transferase (ALT) (U/L), and total cholesterol (TC) (mg/dL). The instrument utilizes Lambert-Beer law and adapts the principle of absorption spectroscopy or the transmission turbidimetry method. Through instructions of the embedded processor in the analyzer, the blood serum will be distributed into the reagent preloaded cuvettes of the rotor to make chemical reactions with test reagents, and color changes will be monitored photometrically by the analyzer. The microprocessor then calculates the analytes’ concentrations to achieve the testing purpose. Briefly, 100 µL of the serum sample was added to each rotor and loaded into the instrument for analysis. For Avian rotors, there was sufficient serum volume (100µL) per bird so that all bird serum samples at each timepoint were run on the Avian rotor. Then we proceeded to run the Liver rotor panel. As such, for the Liver rotors, the volume of some bird serum samples of specific timepoints was technically insufficient (<100 μL), causing the exclusion of these samples from the assay. After all samples were analyzed, the files were exported from the instrument and organized using Microsoft Excel software before further statistical analysis.

### Statistical analysis

Data were analyzed in GraphPad Prism v10.2 (GraphPad, La Jolla, CA). The differences of blood analyte concentrations between lines (HAS male vs. LAS male; HAS female vs. LAS female) and male/female of the same line (HAS male vs. HAS female; LAS male vs. LAS female) were assessed by two-way ANOVA followed by Tukey’s post hoc test. Statistical significance was set at *p* < 0.05.

## Results

### Blood serum inflammatory and nutritional markers diverged between HAS and LAS

**Fig. 1:**
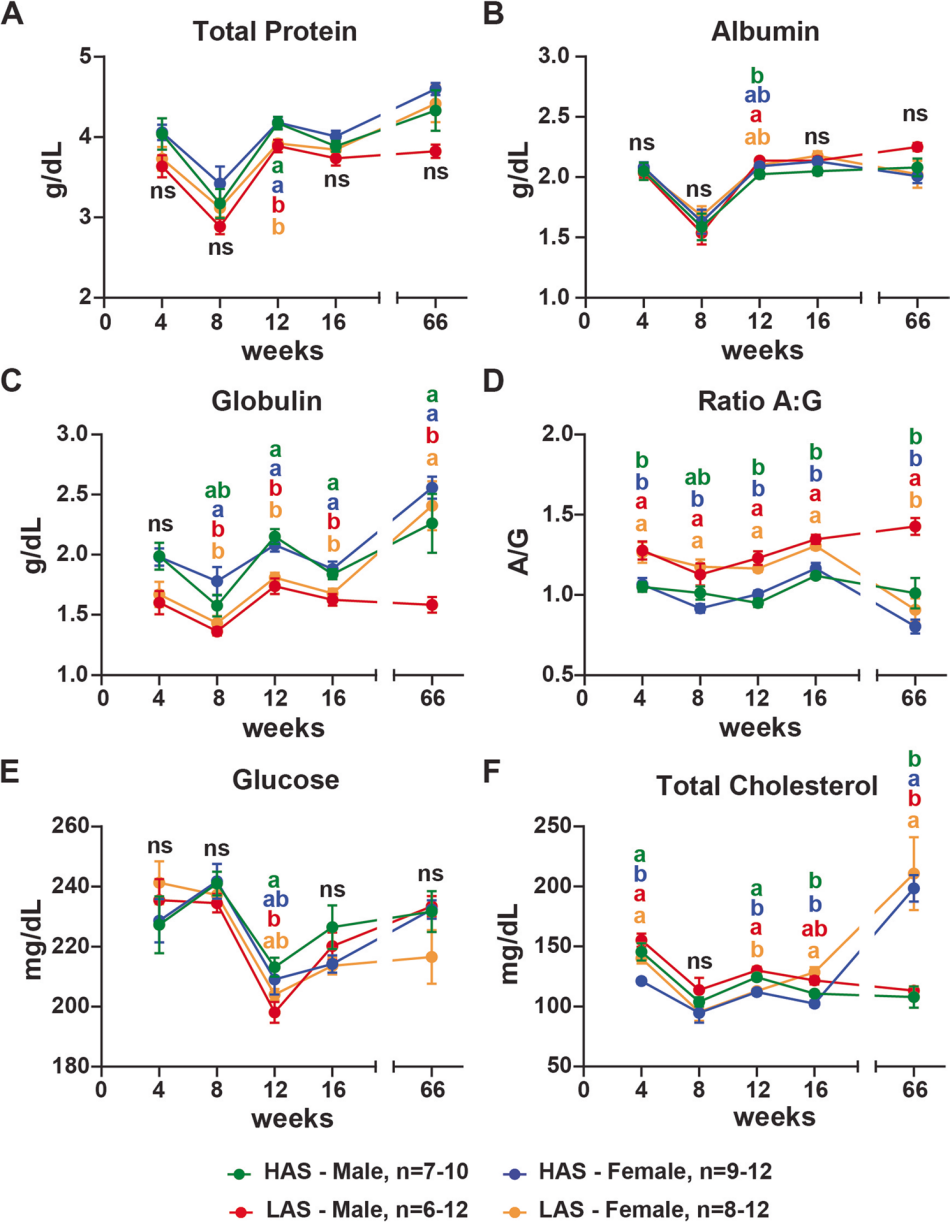
HAS chickens had higher inflammatory markers and different nutritional profiles compared with LAS chickens. Serum was analyzed using MicroChem II with avian or liver rotors as described in Methods for total protein (**A**), albumin (**B**), globulin (**C**), inflammatory marker of albumin levels divided by globulin levels (Ratio A: G) (**D**), glucose (**E**), and total cholesterol (**F**) levels of chickens at 4, 8, 12, 16, and 66 weeks of age in males and females from high (HAS) and low (LAS) antibody lines. n represents the number of chickens utilized in the assays in this study; graphs show mean ± SEM; Data points within a timepoint having no common letter differ significantly at a *p* < 0.05

Serum levels of TP and albumin were similar in both groups at all time points except at 12 weeks, when HAS had higher levels of TP than LAS (p-values: females 0.0442; males 0.473), and albumin levels were higher in LAS males than HAS males (p-values: 0.0382) (Fig. 1A and B). Globulin levels were higher in the HAS than LAS at 12 weeks (p-values: females 0.0051; males 0.0001) and 16 weeks (p-values: females 0.0313; males 0.0395); however, at 8 weeks, only HAS females had higher globulin than LAS (p-value: 0.0235) (Fig. 1C). For the ratio albumin: globulin (ratio A: G), an important inflammatory marker, HAS females, from 4 to 16 weeks of age (p-values: 4 weeks 0.0331; 8 weeks 0.0011; 12 weeks 0.0010; 16 weeks 0.0050), and HAS males, at 4 (p-value: 0.0273), 12 (p-value: <0.0001), and 16 weeks of age (p-value: <0.0001), had lower values than LAS (Fig. 1D). Additionally, at 66 weeks, LAS females had similar globulin levels and ratio A: G to HAS, and were different from LAS males (p-values: LAS females - globulin 0.0035; ratio A: G < 0.0001; HAS males – globulin 0.0303; ratio A: G 0.0005) (Fig. 1C and D). These results indicate line and sex-specific differences in serum protein levels regulation. All TP, albumin, and globulin levels were in the physiological range.

Several markers, including TP, albumin, glucose, and total cholesterol (TC) were used to evaluate the nutritional status through the blood serum biochemistry profile. In this study, both lines had similar levels of glucose, except at 12 weeks, when LAS males had lower glucose levels than HAS males (p-value: 0.0477) (Fig. 1E). For TC, at 4 weeks, HAS females had lower levels than HAS males (p-value: 0.0066) and LAS females (p-value: 0.0382) and males (p-value: 0.0004). In addition, HAS and LAS females’ TC levels were lower than their male counterparts at 12 weeks (p-values: HAS 0.0093; LAS 0.0002). At 16 weeks, HAS females and males had lower levels of TC than LAS females (p-values: females < 0.0001; males 0.0026). Then, at 66 weeks, HAS and LAS females showed higher TC levels compared to HAS and LAS males (p-values: HAS 0.0038; LAS 0.0009) (Fig. 1F). Taken together, the TP, albumin, glucose, and TC results were all within physiological parameters, indicating that all chickens were in a healthy nutritional status.

### HAS and LAS had divergent blood serum electrolyte profiles

**Fig. 2:**
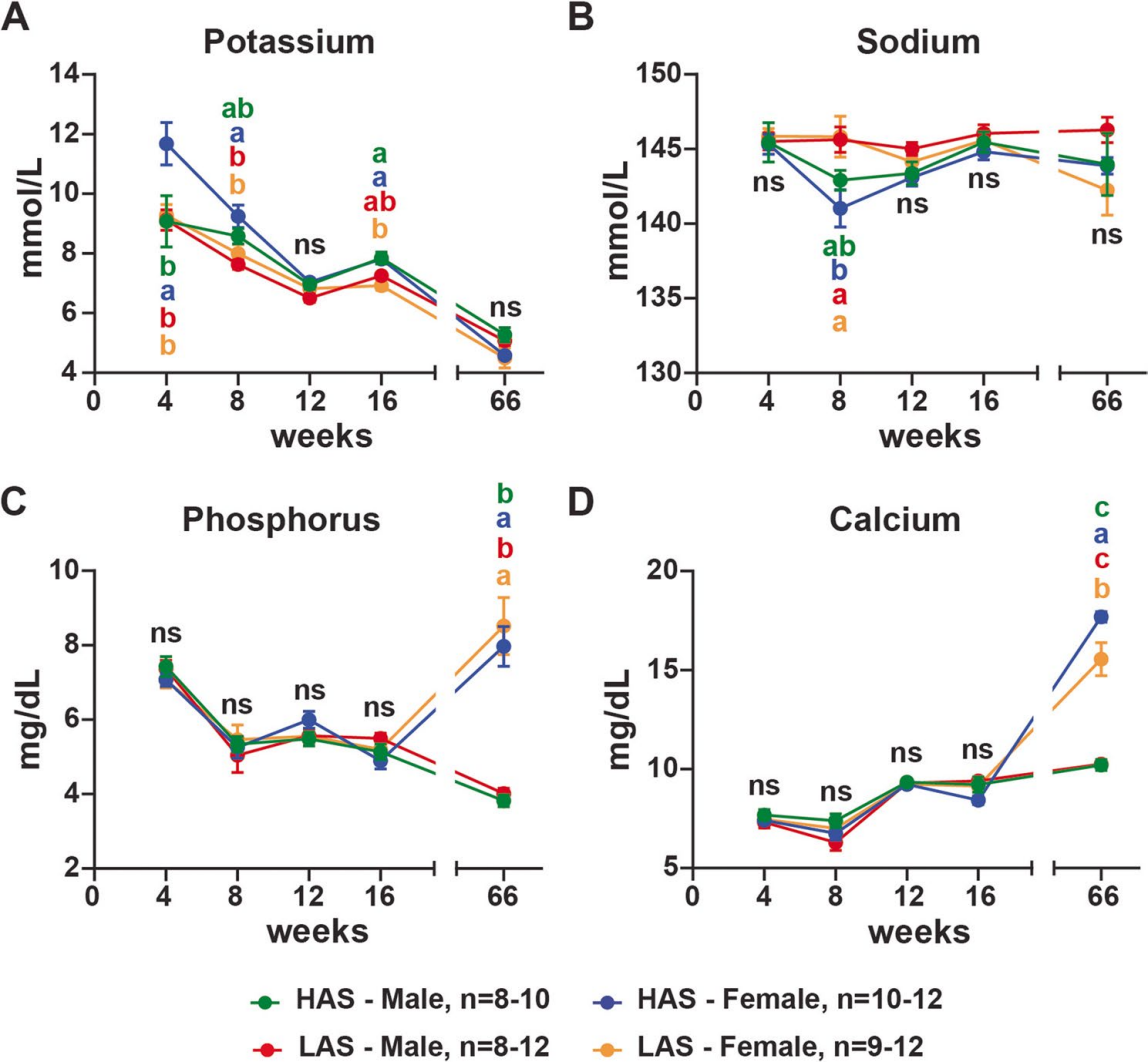
HAS and LAS chickens had divergent serum electrolyte profiles. Serum was analyzed using MicroChem II with avian rotors as described in Methods for potassium (**A**), sodium (**B**), phosphorus (**C**), and calcium (**D**) levels of chickens at 4, 8, 12, 16, and 66 weeks of age in males and females from high (HAS) and low (LAS) antibody lines. n represents the number of chickens used in the assays in this study; graphs show mean ± SEM; Data points within a timepoint having no common letter differ significantly *p* < 0.05

Serum electrolyte concentrations can be used to identify underlying health issues, such as kidney and muscle damage, and acid-base imbalances [20]. At 4 weeks, HAS females had higher potassium levels than other groups (p-values: HAS males 0.0382; LAS females 0.0279; LAS males 0.0266), but similar levels of sodium, phosphorus, and calcium (Fig. 2). At 8 and 16 weeks of age, the profile of potassium diverged, with HAS females having higher potassium levels than LAS females (p-values: 8 weeks 0.0211; 16 weeks 0.0007), though HAS and LAS had similar levels of potassium at 12 weeks (Fig. 2A). Both lines had similar levels of phosphorus and calcium during this period (Fig. 2C and D). At 8 weeks, HAS females had decreased sodium levels compared to LAS females and males (p-values: females 0.0188; males 0.0383) (Fig. 2B). Serum sodium levels were similar among HAS and LAS groups in weeks 4, 12, 16, and 66 (Fig. 2B). At 66 weeks, HAS and LAS females had higher phosphorus and calcium levels than their male counterparts (p-values: phosphorus – HAS < 0.0001; LAS < 0.0001; calcium – HAS < 0.0001; LAS < 0.0001) (Fig. 2C and D). Moreover, HAS females had higher calcium levels than LAS females (p-value: 0.0125) (Fig. 2D). Our results suggest a divergent electrolyte profile between the HAS and LAS lines.

### HAS chickens exhibited a serum biochemistry profile of muscle damage, without associated kidney damage

**Fig. 3:**
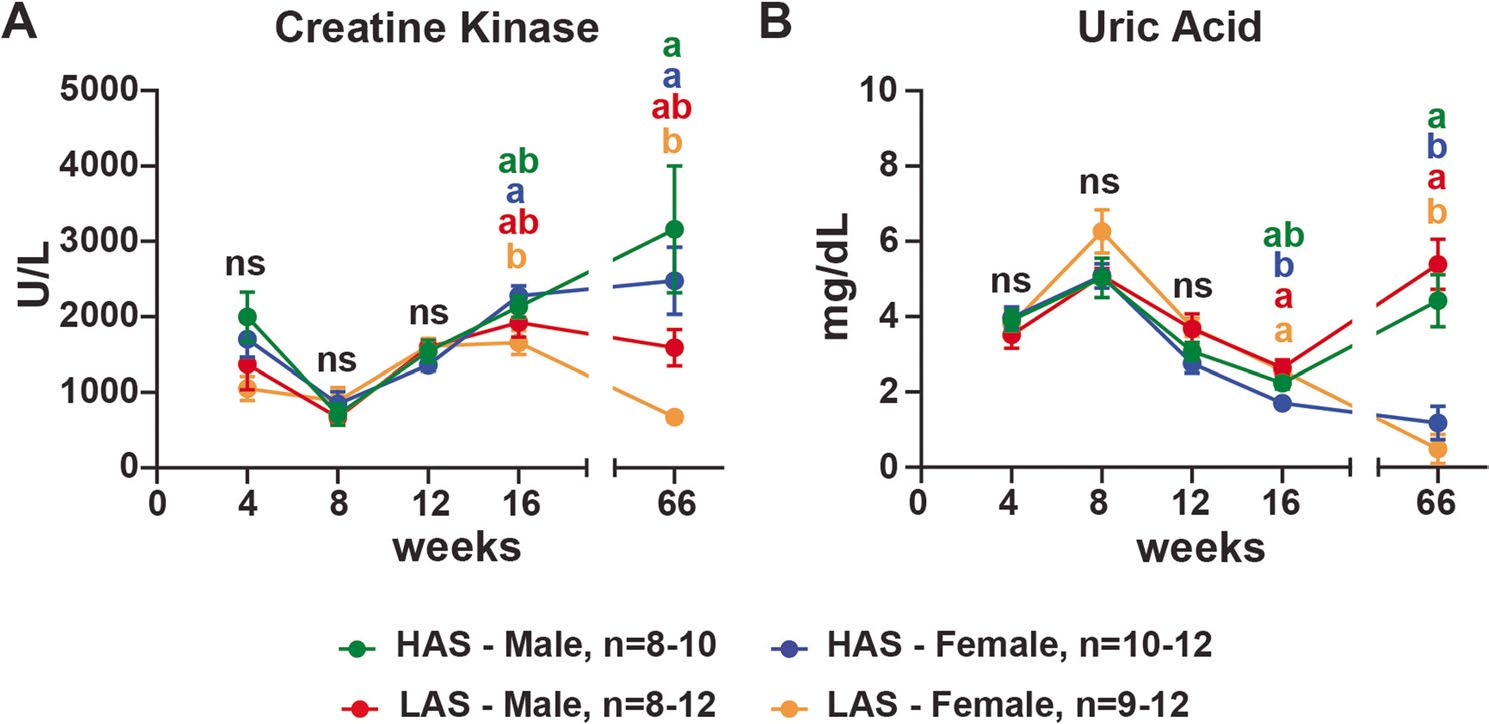
HAS chickens had the serum biochemistry profile of muscle damage without associated kidney damage. Serum was analyzed using MicroChem II with avian rotors as described in Methods for creatine kinase (**A**), and uric acid (**B**) levels of chickens at 4, 8, 12, 16, and 66 weeks of age in males and females from high (HAS) or low (LAS) antibody lines. n represents the number of chickens used in the assays in this study; graphs show mean ± SEM; Data points within a timepoint having no common letter differ significantly, *p* < 0.05

Creatine kinase (CK) is an enzyme primarily found in muscle tissue, such as skeletal muscles and the heart [20]. Increased levels of this enzyme in the serum are mainly associated with skeletal muscle injury. At 16 weeks, HAS females had higher CK levels than LAS females (p-value: 0.0309). At 66 weeks, HAS levels of this enzyme further increased, while in LAS, there was a decrease in CK levels (p-values: females 0.0304; males 0.0946) (Fig. 3A).

Serum concentration of uric acid is mainly associated with kidney function [21]. HAS females had a lower uric acid concentration at 16 weeks than LAS females and males (p-value: females 0.0022; males 0.0013) (Fig. 3B). Additionally, at 66 weeks, HAS and LAS females had lower concentrations of uric acid than their male counterparts (p-values: HAS 0.0012; LAS < 0.0001) (Fig. 3B). All four group values for uric acid were within the physiological normal range.

### HAS and LAS lines diverged in the liver function profile after 8 weeks of age

**Fig. 4:**
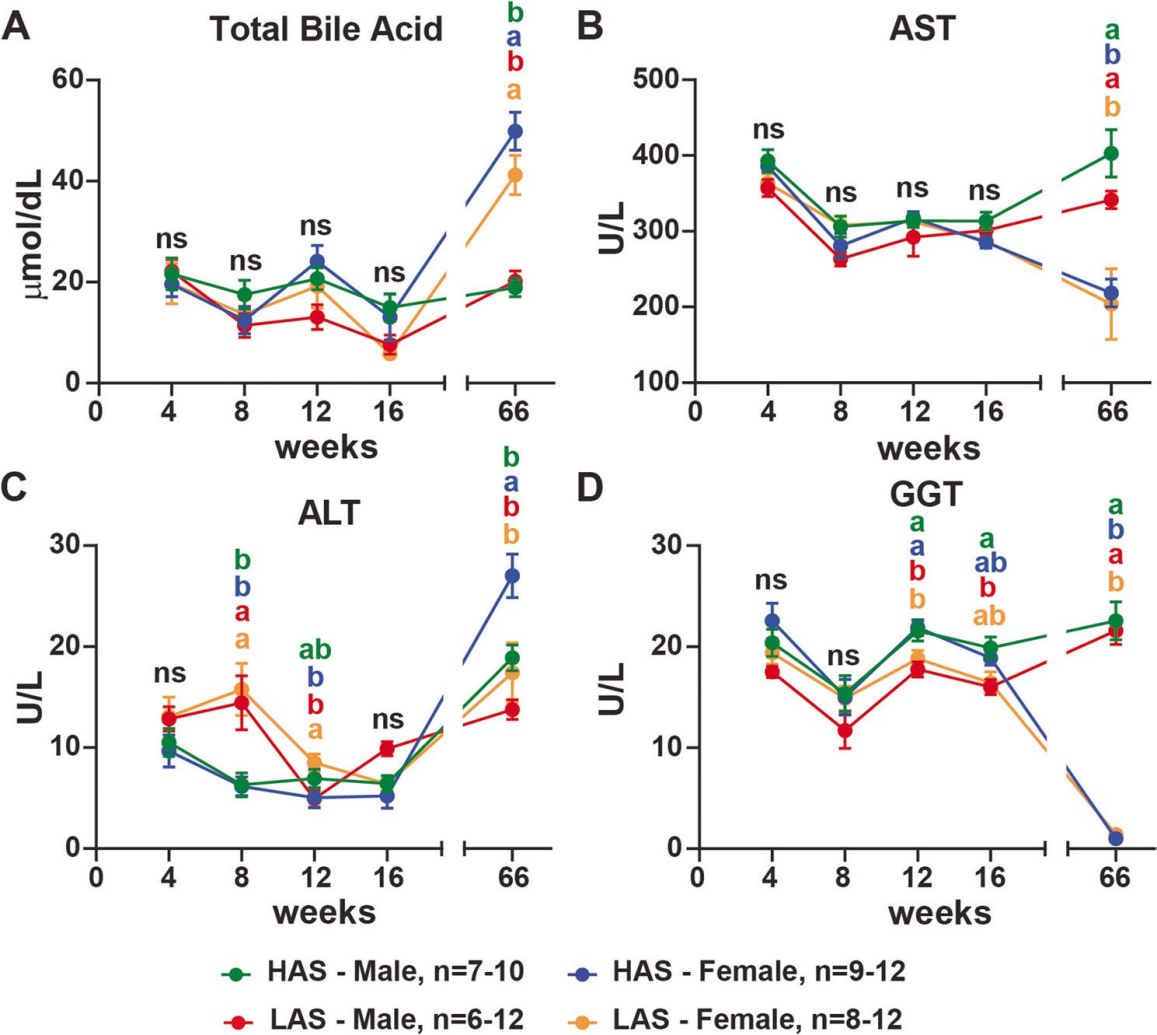
HAS and LAS chicken lines had divergent liver function profiles after 8 weeks of age. Serum was analyzed using MicroChem II with avian or liver rotors as described in Methods for total bile acid (**A**), aspartate transferase (AST) (**B**), alanine transferase (ALT) (**C**), and gamma-glutamyl transferase (GGT) (**D**) levels of chickens at 4, 8, 12, 16, and 66 weeks of age in males and females from high (HAS) or low (LAS) antibody lines. n represents the number of chickens used in the assays in this study; graphs show mean ± SEM; Data points within a timepoint having no common letter differ significantly, *p* < 0.05

Liver function can be evaluated by the measurement of its products, such as total bile acids (TBA) or its enzymes, aspartate transferase (AST), alanine transferase (ALT), and gamma-glutamyl transferase (GGT). In our study, TBA levels were similar between the groups at 4, 8, 12, and 16 weeks. At 66 weeks, TBA levels in females were higher than in males (p-values: HAS < 0.0001; LAS 0.001) (Fig. 4A). For AST, similar levels among the groups were seen at 4, 8, 12, and 16 weeks. HAS and LAS females’ AST levels were lower than males from both lines at 66 weeks (p-values: HAS 0.008; LAS 0.0106) (Fig. 4B). ALT levels were similar between the groups at 4 and 16 weeks. At 8 weeks, ALT levels diverged between the lines; LAS had higher ALT levels than HAS (p-values: females 0.0039; males 0.0319). LAS females had higher ALT concentration than the LAS males (p-value: 0.0470) and HAS females (p-value: 0.0397) at 12 weeks, whereas HAS females had higher ALT concentrations than all other groups at 66 weeks (p-values: HAS males 0.0450; LAS females 0.0080; LAS males 0.0001) (Fig. 4C). All groups had similar GGT concentrations at 4 and 8 weeks. By 12 weeks, HAS birds had higher levels than LAS (p-values: females 0.424; males 0.0278), but at 16 weeks, only HAS males had higher GGT levels than the LAS males (p-value: 0.0360), with the two female lines intermediate. At 66 weeks, HAS and LAS females’ GGT levels were lower than HAS and LAS males (p-values: HAS < 0.0001; LAS < 0.001) (Fig. 4D). All chickens had liver enzyme levels within the physiological range. Our data suggest a divergent liver function profile of HAS and LAS between 4 and 16 weeks, though at 66 weeks, the liver profiles of females from both lines diverged from their respective males.

## Discussion

Albumin is the largest protein fraction and is important for maintaining osmotic pressure and the transport of metabolites. Decreased albumin concentrations can indicate malnutrition, chronic liver or kidney diseases, and/or inflammation [21]. Globulin is a group of blood pro-inflammatory proteins, which includes alpha-globulins and beta-globulins, which are considered acute phase proteins, and gamma-globulins, which are elevated in chronic inflammatory conditions, and include immunoglobulins [20, 21]. Total protein (TP) is the sum of albumin and globulin concentrations, and the ratio A: G is a marker for inflammatory diseases (lower values are related to increased globulin levels) [8, 22]. The pattern of higher globulin concentrations and persistently lower A: G ratios in the HAS birds is consistent with long-term divergent selection for antibody response to SRBC. Blood serum globulin fractions are dominated by immunoglobulins and other immune-associated proteins. Recent studies involving these lines report correlated changes in immune organ gene expression and humoral immune indices [23]. Together, these results suggest that the elevated globulin and reduced A: G ratio observed are biologically consistent with an enhanced antibody profile rather than associated with nutritional or inflammatory conditions [3, 23]. The transient changes in TP and albumin at 12 weeks and the sex-specific pattern of higher globulin in LAS females at 66 weeks support that age, reproductive status, and sex can play a role in the regulation of serum protein profiles [24, 25].

Previous studies [2, 14] utilizing the HAS and LAS lines have shown that the divergent selection is associated with coevolution in the gut microbiome and identified specific microbial taxa whose abundance can accurately predict the chicken line as HAS or LAS. One of the differences is that HAS showed enrichment in modules for microbial riboflavin and lysine biosynthesis, which are associated with immune cell function and antibody response [2], compared with LAS. Utilization of nutrients by the microbiota can alter the nutrient availability for the host and, as a consequence, modify the circulating metabolites detectable in serum [19]. The gut microbiota is an important key regulator of nutrient absorption, metabolite production, and immune regulation. Previous studies have shown that alterations in the diet can alter the serum biochemistry levels of glucose, triglycerides, and TC in chickens [26–28]. Our results showed HAS and LAS had divergent levels of glucose at 12 weeks and TC at 16 weeks without a diet change, suggesting a consequence of the divergent coevolution with the gut microbiome.

The physiological interval for serum potassium in chickens can vary according to age, breed, and environmental factors, based on multiple studies, the physiological range of serum potassium for laying hens is 0–5.1 mmol/L [21, 29–32]. In our study, most chickens in both lines had potassium levels considered higher than the physiological range at all timepoints. High levels of serum potassium are usually caused by kidney diseases or high-potassium diets [21], neither of which were present in this study. This suggests that the high potassium levels were a result of the divergent selection for SRBC antibody. The serum potassium levels of these chickens could have been the consequence of the coevolution of the gut microbiome [2, 14]. One example of how the microbiome can alter the blood ionic profile is that butyrate, a short-chain fatty acids (SCFA) produced by colonic bacterial fermentation, can activate potassium secretion on the apical membrane of large intestinal mucosal cells, altering the blood levels of potassium [33]. In poultry, microbiome differences have been linked to altered SCFA, bile acid metabolism, and serum biochemical markers [17, 18]. The microbiome production of SCFA, such as butyrate and, to a lesser degree, propionate, can inhibit dendritic cells, T helper cells, and B cells’ functions [34–38], leading to reduced immunoglobulin production. Selection for immune traits can shape gut microbiota composition through mucosal immunity or systemic inflammation, which in turn alters host metabolic signatures [3, 7, 19]. We previously demonstrated that LAS cecal microbiome metabolic activity was different from HAS. Indeed, the LAS microbiome presented enrichment in the L-tryptophan biosynthesis, carbon metabolism, and degradation process pathways [7]. Furthermore, higher *Ruminococcaceae* abundance [7], a genus which belongs to a known group of butyrate producers of human colon microbiota [39, 40], was higher in LAS than HAS. Taken together, our data suggest that the divergent selection for high and low antibody titers influences the gut microbial taxa as well as serum potassium profiles.

Although increased serum CK concentrations are usually associated with muscle tissue damage, levels may increase with physiological exercise and stress. Dabbert and Powell (1993) showed that wild mallards captured by entanglement nets had six times higher CK concentration than the group with minimal handling, implying that stress played a role in the increased levels of CK [41]. Additionally, in cats, the increase of serum CK can be associated with anorexia, because it increases muscle catabolism [42]. HAS chickens have higher ATP production, immune cell motility and inflammation in the jejunum than LAS, suggesting a “primed” system with higher metabolism [43]. The overproduction of immunoglobulins is known as hypergammaglobulinemia, commonly caused by liver disease, acute or chronic inflammation, and autoimmune disorders, which can cause systemic amyloidosis. Systemic immunoglobulin light chain amyloidosis is a metabolic disorder caused by the deposit of abnormal proteins in the extracellular matrix of various organs and tissues [44]. The result is progressive organ dysfunction by the disruption of tissue architecture. In humans, systemic amyloidosis most commonly causes glomerulonephritis and cardiomyopathy [44–46]. In chickens, it is referred to as AA amyloidosis and can be induced by repeated inflammatory stimulations via vaccinations with oil-emulsified bacterins, leading to amyloid deposition in the pectoral muscle in mature chickens [47, 48]. The HAS chickens at 66 weeks had CK levels higher than the physiological range (65–2340 U/L) [29], consistent with an increase in muscle metabolism or damage, suggesting that long-term selection for high antibody response to SRBC may lead to myopathy.

Uric acid is synthesized in the liver and excreted through kidney filtration, and ~90% is excreted via tubular secretion independent of the urine flow rate. It is the major end product of nitrogen metabolism in birds, and its serum concentration increases are associated with kidney damage when the impairment exceeds ~70% of its original function [20]. However, uric acid levels in both chicken lines were within the physiological range (0–13 mg/dL) [21], suggesting the high levels of immunoglobulins in HAS did not cause kidney damage.

The liver has numerous physiological functions, including the synthesis of immune response proteins, nutrient metabolism, and detoxification [8]. Alteration of the blood serum levels of liver enzymes are associated with changes in hepatic function, which may result from nutritional imbalances, toxin exposure, metabolic stress, and infectious diseases [8, 49]. The high activity of AST in the liver, skeletal muscle, heart, brain, and kidneys may explain the variation of this enzyme with age of birds, and is consistent with HAS and LAS males; older broiler chickens present a pattern of AST levels increasing as they age [50]. GGT is found in the liver, pancreas, and kidney, but its serum elevation is specific to liver damage because kidney GGT is excreted in the urine [21, 51]. Bowes et al. (1989) reported that with age, male White Leghorn and broiler chickens had increased calcium, sodium, TP, and albumin, while potassium and AST levels were decreased with age [52]. In the present study, we observed a similar pattern with age: i.e., an increase in calcium and TP levels, and a decrease in potassium in all groups, but only females had decreased levels of AST. Additionally, egg production intensity can significantly impact liver function, causing considerable fluctuation of the blood serum levels of liver enzymes in laying hens [53]. Also, laying birds have lower levels of uric acid and increased serum concentrations of calcium, phosphorus and TC [29, 52, 54]. The 66-week-old females of both lines had lower levels of uric acid, GGT, and AST, but higher serum concentrations of calcium, phosphorus, TBA, and TC, consistent with egg-laying bird serum biochemistry profiles.

The divergent selection in the antibody production of HAS and LAS lines resulted in correlated blood serum biochemistry profiles. Most of the biochemical analyte levels were within the physiological range, suggesting that the divergent profiles are related to the specialization of each chicken line and that we can use blood serum biochemical profiles to evaluate animal productivity. Previous studies showed that fat and lean lines of broiler chickens presented divergent blood serum biochemistry profiles [55] and low and high meat pH levels were correlated to the blood serum lipid profiles of broiler chickens [56]. Additionally, studies showed that alteration in the diet of laying hens can alter their serum biochemistry profile and consequently cause alteration in their egg composition [57, 58]. For instance, the supplementation of calcium and cholecalciferol in the regular diet can lead to a decrease in serum triglycerides and a simultaneous increase in serum albumin, serum immunoglobulins, and eggshell quality [57]. These data show the potential of using serum biochemistry analysis as a prediction factor for poultry production performance.

It is worth highlighting that the present study included chickens from 4 to 16 weeks of age and then at 66 weeks of age, in which we observed age-specific differences in some of the measured serum biochemical variables. Including an increase of AST and a decrease of potassium levels in older chickens, which warrants future studies that utilize chickens between 16 and 66 weeks of age. The addition of chicken age groups between 16 and 66 weeks of age will likely help to elucidate how selection for divergence in blood antibody titers affects long-term patterns in separation of serum biochemical profiles.

## Conclusions

The present study demonstrates that chickens subjected to long-term selection for antibody response to SRBC display significantly altered serum biochemistry profiles. Age and reproductive status also contributed to the blood serum biochemistry profiles of these selected lines, which originated from a common founder population. More specifically, HAS had higher levels of total protein, globulins, potassium, and CK than LAS. Additionally, the divergent chicken lines had distinct liver enzyme profiles. Our results provide evidence that the serum biochemistry profile can be used as a tool to understand how the selection for humoral immune response can affect the physiology of chickens. The results presented herein enhance the understanding of the different serum biochemistry markers that give insights into how the immune system influences the nutritional and health status of individual chickens. Such insights increase understanding of the interplay between the immune system, protein and lipid metabolisms, and the gut microbiome.

## Data Availability

All data generated or analyzed during this study are available upon reasonable request.

## Abbreviations

ALT: Alanine transferase
AST: Aspartate transferase
CK: Creatine kinase
GGT: Gamma-glutamyl transferase
HAS: High antibody chicken line
LAS: Low antibody chicken line
Ratio A: G: ratio albumin: globulin
SCFA: Short-chain fatty acids
TBA: Total bile acids
TC: Total cholesterol
TP: Total protein
UA: Uric acid
